# Photocatalytic Performance Evaluation of Titanium Dioxide Nanotube-Reinforced Cement Paste

**DOI:** 10.3390/ma13235423

**Published:** 2020-11-28

**Authors:** Junxing Liu, Hyeonseok Jee, Myungkwan Lim, Joo Hyung Kim, Seung Jun Kwon, Kwang Myong Lee, Erfan Zal Nezhad, Sungchul Bae

**Affiliations:** 1 Department of Architectural Engineering, Hanyang University, 222, Wangsimni-ro, Sungdong-gu, Seoul 04763, Korea; liujx128119@hanyang.ac.kr (J.L.); wlgustjr01@gmail.com (H.J.); 2 Department of Architecture Engineering, Songwon University, Gwangju 61756, Korea; limmk79@naver.com; 3 Korea Conformity Lab, Construction Technology Research Center, Construction Division, Seoul 08503, Korea; kjhmole@kcl.re.kr; 4 Department of Civil and Environmental Engineering, Hannam University, Daejeon 306-791, Korea; jjuni98@hnu.kr; 5 Department of Civil, Architectural, and Environmental System Engineering, Sungkyunkwan University, Gyeonggi-Do 16419, Korea; leekm79@skku.edu; 6 Department of Chemical and Biomedical Engineering, University of Texas at San Antonio (UTSA), San Antonio, TX 78249, USA; erfan.zalnezhad@utsa.edu

**Keywords:** photocatalysis, titanium dioxide nanotube, anatase TiO_2_, hydration products, cement paste

## Abstract

Considering the increase in research regarding environmental pollution reduction, the utilization of cementitious material, a commonly used construction material, in photocatalysts has become a desirable research field for the widespread application of photocatalytic degradation technology. Nano-reinforcement technology for cementitious materials has been extensively researched and developed. In this work, as a new and promising reinforcing agent for cementitious materials, the photocatalytic performance of titanium dioxide nanotube (TNT) was investigated. The degradation of methylene blue was used to evaluate the photocatalytic performance of the TNT-reinforced cement paste. In addition, cement paste containing micro-TiO_2_ (m-TiO_2_) and nano-TiO_2_ (n-TiO_2_) particles were used for comparison. Moreover, the effect of these TiO_2_-based photocatalytic materials on the cement hydration products was monitored via X-ray diffraction (XRD) and thermogravimetric analysis (TG). The results indicated that all the TiO_2_ based materials promoted the formation of hydration products. After 28 days of curing, the TNT-reinforced cement paste contained the maximum amount of hydration products (Ca(OH)_2_). Furthermore, the cement paste containing TNT exhibited better photocatalytic effects than that containing n-TiO_2_, but worse than that containing m-TiO_2_.

## 1. Introduction

TiO_2_ and its nanostructured materials have been extensively investigated and applied in several fields, including photocatalysis [1,2], Li-ion batteries [3,4], and dye-sensitized solar cells [5,6] owing to their low cost, high chemical stability, nontoxicity, and super-hydrophobicity [1,7]. Typically, there are three polymorphs of TiO_2_: anatase, rutile, and brookite. Anatase TiO_2_ exhibits higher photocatalytic activity than the other phases, owing to its higher surface area, surface adsorption capacity, and lower charge carrier recombination rate [8]. Therefore, anatase TiO_2_ is the most widely used photocatalyst capable of removing atmospheric pollutants, such as nitrogen oxides (NO_x_), volatile organic oxides (VOCs), and nonvolatile organic residues, via redox reactions on the catalyst surface under natural sunlight (usually in the UV range) [9]. Hamidi et al. [10] suggested that photocatalytic efficiency is a complicated aspect influenced by several factors and concluded that the five factors that most affect the efficiency of photocatalytic processes are: (a) effective absorption of photon energy, (b) fast charge separation after absorption of photon energy to prevent electron–hole recombination, (c) separation of products from the surface of the photocatalyst, (d) long term photocatalyst stability, and (e) the redox potential of the electron–hole pair should be compatible with the redox potentials of the donor or acceptor species. These factors only take into account the TiO_2_-based materials themselves. However, pure TiO_2_ micro- and nanostructures are rarely directly combined into bulk materials for use; therefore, a convenient way to overcome this drawback is to attach the TiO_2_-based material in/on different support materials [11].

Nano-reinforcement technology plays a critical role in a novel research direction in the field of construction and building materials [12]. Among the materials used in civil and architectural fields, cementitious materials, such as concrete, are considered to be one of the most popular materials used and consumed. In addition, the rich pore structure and chemical stability of cement hydrate products give cementitious materials a strong binding capacity and good processability [13]. These characteristics make cementitious materials suitable supports for loading nanomaterials [14]. On one hand, some literature reports suggest that nanomaterials have a reinforcement effect on cementitious materials, such as mechanical strength, durability, and ductility, and can accelerate the hydration reaction [15,16,17]. Chen et al. [18] found that after 28 days of curing, the compressive strength of the cement mortar increased from 40 MPa to 50 MPa with increasing the amount of nano-TiO_2_ (up to 10% of cement weight). Li et al. [19] studied that the size effect of TiO_2_ on the properties of cementitious materials and found that the nucleation effect and reinforcement of the mechanical properties of cement were more significant with smaller size TiO_2_ (10 nm) than larger size TiO_2_ (15 nm). Furthermore, previous studies [18,20] have shown that the incorporation of TiO_2_ into cementitious materials can improve its mechanical strength owing to the ability of TiO_2_ to accelerate the hydration process as well as the pore-refining effect.

On the other hand, the incorporation of nanomaterials into cementitious materials offers some novel features, such as thermal resistance [21], self-sensing [22], self-healing [23], and self-cleaning [24]. The incorporation of photocatalysts, such as TiO_2_-based materials, into cementitious materials, is undoubtedly one of the most significant topics in the field of environmentally friendly building materials since the addition of photocatalysts contributes to the air-purifying, self-cleaning, self-sterilizing, and anti-fogging properties of the construction materials [25]. The advantage of this material is that, apart from the addition of TiO_2_ particles to the building materials, only sunlight, oxygen, and water are required [26]. Seo et al. [27] researched that the NO removal rate for the photocatalytic cement-based materials and noted that the smaller size TiO_2_ particles projected the higher NO absorption and removal rate in the dry condition. In addition, Zhang et al. [28] pointed out that particle size was important in nanocrystalline TiO_2_-based catalysts mainly by influencing the kinetics of e^−^/h^+^ pair recombination. For methods of using photocatalytic materials in cementitious materials, besides adding the photocatalyst to the cementitious material, another way to provide a photocatalytic effect to the cementitious material is to attach the photocatalyst to the surface of the cementitious material as a thin film by coating. Feng et al. [14] investigated the photocatalytic performance of cement paste utilized smear method to prepare TiO_2_ film on the cement paste surface, and the result showed that the photocatalytic cement paste could nearly completely discolor 10 mg/L Rhodamine B, methylene blue, and methylene orange solution in 50 min under UV light. In addition, Park et al. found that the incorporation of nano-TiO_2_ into cement paste enhances the neutron shielding performance of the cement paste via Monte Carlo simulation at the thermal, slow, and intermediate neutron energy levels [29]. In summary, TiO_2_ cannot only reinforce cementitious materials in terms of strength and hydration processes but can also add photocatalytic properties to cement-based materials. In 1998, Kasuga et al. [30] first reported the synthesis of TNT through a hydrothermal method by making use of anatase phase TiO_2_ and revealed that TNT exhibits relatively larger specific surface areas than typical TiO_2_ nanopowders. Therefore, TNT has been widely utilized in various applications, including photocatalysis [31,32], solar cells [33], and electrochromic devices [34]. According to our previous research [35], the incorporation of TNT into cementitious materials enhanced the compressive strength and flexural strength by 11.7% and 23.5%, respectively, after 28 days at low addition dosages (0.5% of cement weight) and accelerated the hydration reaction rate of the cementitious materials. This proved the possibility of using TNT as a nano-reinforcement agent for cement-based materials. However, there have been few studies on the photocatalytic properties of TNT in cementitious materials.

Thus, in this study, we aimed to investigate the photocatalytic performance of TNT-reinforced cement paste as well as cement paste blended with m-TiO_2_ and n-TiO_2_ particles for comparison. The TNT was synthesized through a hydrothermal method, and the surface area of TNT was checked by Brunauer–Emmett–Teller (BET) surface area analysis. The photocatalytic effects of the different TiO_2_-based materials in the hardened cement were evaluated by determining the changes in methylene blue (MB) concentration by ultraviolet-visible (UV-vis) spectroscopy. Transmission electron microscopy (TEM) was used to inspect the morphology of the TiO_2_ based materials. The effects of the TNT and TiO_2_ particles on the hydration products were evaluated via X-ray diffraction (XRD) and thermogravimetric analysis (TG).

## 2. Experimental Procedures

### 2.1. Raw Materials

Ordinary Portland cement (OPC), provided by Sungshin Co. Ltd., Seoul, Korea, was used to produce the cement paste. The chemical composition, measured by X-ray fluorescence spectroscopy (XRF, ZSX Primusll, Rigaku, Tokyo, Japan), is shown in Table 1. The microsized anatase titanium (IV) dioxide (m-TiO_2_) used in this work was purchased from FUJIFILM Wako Pure Chemical Corporation, Tokyo, Japan. Anatase titanium (IV) oxide nanopowder (n-TiO_2_), purchased from Sigma Aldrich (Seoul, Korea), was used to synthesize TNT. The physical properties of the different TiO_2_ powders are summarized in Table 2.

### 2.2. Hydrothermal Synthesis of TNT

**Figure 1:**
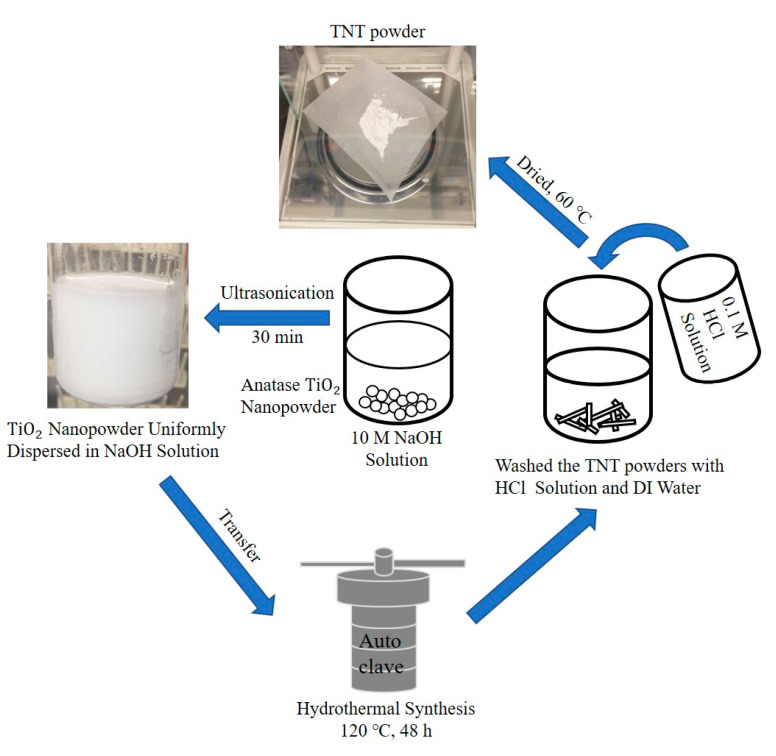
The titanium dioxide nanotube (TNT) hydrothermal synthesis process.

The TNT was synthesized via the hydrothermal method developed by Kasuga et al. [30,36]. This method is a simple experimental route to obtain a tubular TNT structure [37]. Figure 1 illustrates the hydrothermal TNT synthetic process, detailed as follows: 1.0 g of TiO_2_ anatase nanopowder was mixed with 50 mL of 10 M NaOH solution. Next, the mixture was sonicated for 30 min in a 20 s work cycle using an ultrasonic liquid processor (Q700 Sonicator, Qsonica, Newtown, CT, USA, 20 kHz, amplitude: 50%). Then, the homogeneous suspension was transferred to a Teflon autoclave to start the hydrothermal synthesis process at 120 °C for 48 h. Subsequently, the obtained product was naturally cooled to room temperature and washed successively with 0.1 M HCl solution and distilled water until the pH was close to neutral. Finally, the precipitate was filtered and dried at 60 °C for 24 h. The morphology and crystal structure of the TNT was monitored by TEM and XRD.

### 2.3. BET Analysis

The surface area of TNT was confirmed via N_2_ adsorption on the 3Flex surface characterization analyzer at 77 K. The specific surface area was calculated by BET Equation (1).

(1) $$\frac{1}{X\left[\left[P_{0}/P\right]-1\right]}=\frac{1}{X_{m}C}+\frac{C-1}{X_{m}C}\left(\frac{P}{P_{0}}\right)$$

where X is the weight of adsorbed N_2_ at certain relative pressure (P/P_0_), X_m_ is the volume of single-layer adsorption capacity of the gas at the standard temperature and pressure (273 K and 1 atm), C is constant.

### 2.4. Preparation of Cement Paste

**Figure 2:**
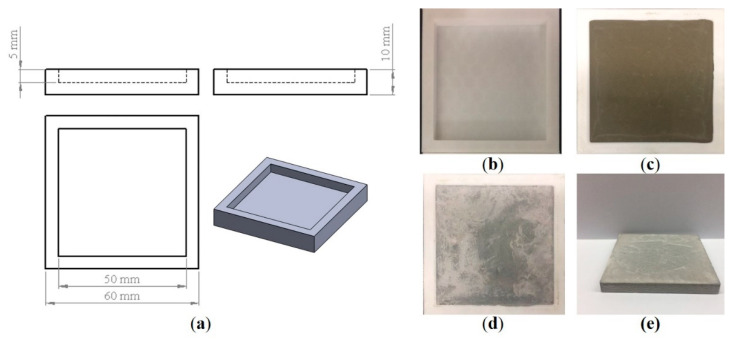
Schematic of the (**a**) three views of the cement mold, (**b**) real product of cement mold, (**c**) the fresh cement paste in the mold, (**d**) hardened cement paste in the mold, and (**e**) cement paste after 1 day of curing and demolding.

Pure OPC paste and cement pastes containing photocatalyst powders were fabricated with a water-to-cement ratio (w/c) of 0.4. The mass content of TiO_2_-based materials (m-TiO_2_, n-TiO_2_, and TNT) was 1 wt% of the mass of cement. Details of the mixed proportion of specimens are shown in Table 3, and the names of the three TiO_2_-based materials were used to refer directly to the different specimens. To prepare the cement paste for testing, the different TiO_2_-based materials were soaked in distilled water and ultrasonicated (Q700 Sonicator, Qsonica, Newtown, CT, USA, 20 kHz, amplitude: 50%) for 15 min to achieve a homogeneous solution before being added to the cement. The homogeneous solution was mixed with cement via a paste mixer (SPS-1, Malcom, Tokyo, Japan) for 12 min. The specimen was demolded after 24 h and cured at 25 °C and 65% relative humidity until being tested. The polylactic acid (PLA) molds used to obtain the specimens (50 mm × 50 mm × 5 mm) were designed using SolidWorks and fabricated with a fused-deposition modeling 3D printer (Guider Ⅱ, Flashforge, Jinhua, China), as shown in Figure 2a. The reason for choosing the shape of the sample shown in Figure 2e was to obtain a relatively large surface that was easy to place and use in the photocatalytic test.

### 2.5. X-ray Diffraction

Phase characterization of the hardened cement pastes containing the nanomaterials was conducted using an XRD spectrometer (D2 PHASER, Bruker, Billerica, MA, USA) equipped with Cu Kα radiation (λ = 1.5406 Å). The measurement covered the angular range (2θ) from 7° to 70° with a step size of 0.01° at 1.5 sec per step. The phase was identified using the DIFFRAC.EVA software (Bruker, Billerica, MA, USA). The XRD samples were prepared by first grinding in a mortar and pestle and then sieved through a 400-mesh sieve.

### 2.6. Thermogravimetric Analysis

To determine the composition of the cement hydrates, TG and first derivative thermogravimetric analysis (DTG) curves were utilized [38]. The test was performed using a HITACHI STA7200 Simultaneous Thermogravimetric Analyzer (Tokyo, Japan) with a sensitivity of 0.2 μg. At the two test times (1 and 28 days), approximately 15 mg of the specimens were crushed and ground to obtain a particle size smaller than 75 μm. The test temperature ranged between 20 and 900 °C with a heating rate of 10 °C/min and an N_2_ atmosphere of 200 mL/min.

### 2.7. Transmission Electron Microscopy

The morphologies of the m-TiO_2_, n-TiO_2_, and TNT were obtained by TEM (JEM2100F, JEOL, Tokyo, Japan). To prepare the samples for TEM, a suitable amount of powder was dissolved in ethanol by ultrasonic vibration, and then a few drops of the sonicated solution were placed on a carbon film Cu grid and dried for 48 h in a desiccator.

### 2.8. Photocatalytic Test by Methylene Blue Degradation

**Figure 3:**
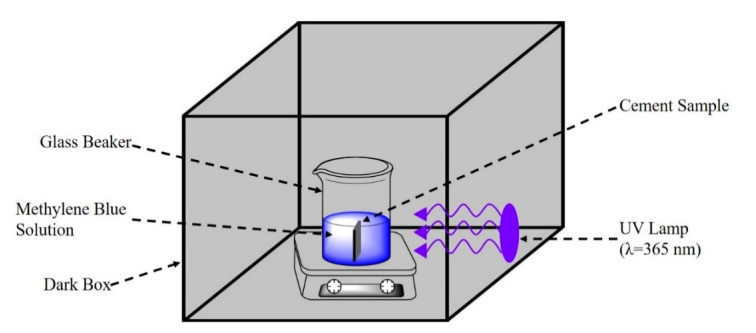
Schematic diagram of the photocatalytic test.

The photocatalytic activities of the prepared specimens (Table 3) were assessed by testing the decomposition of MB as an organic pollutant under UV light (PM-1600UVH, NDT Advance, Saitama, Japan) irradiation. Figure 3 shows a schematic diagram of the test equipment. The experimental conditions were as follows: after curing for 28 days, the hardened cement paste was immersed in 200 mL of MB solution in a 250 mL beaker. During the entire process, the solution was continuously stirred with a magnetic stirrer to keep the concentration of the solution consistent. Before irradiation, the MB solution containing the cement paste specimen was placed in the dark for 1 h to reach an adsorption–desorption equilibrium. Approximately 2 mL of MB solution was collected at 1 h intervals during the 5 h test. The change in MB concentration was monitored by UV-vis spectroscopy (Genesys 180, Thermo Scientific, Waltham, MA, USA) at the characteristic λ_max_ = 665 nm. The wavelength range used in the test was 200–800 nm. The normalized concentration of the photocatalysts was calculated using Equation (2):

Normalized concentration = c/c_0_ (2)

where c represents the concentration of the MB solution at different time intervals, and c_0_ represents the initial concentration of MB solution.

## 3. Results and Discussion

### 3.1. Characteristics of the Raw Materials

**Figure 4:**
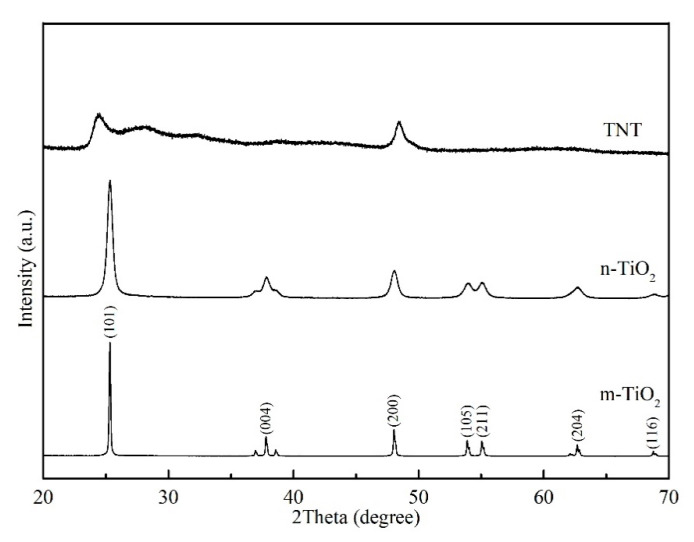
Comparison of the XRD patterns of m-TiO_2_, n-TiO_2_, and TNT.

The crystallographic structure and relative crystallinity of the materials were identified via XRD. The XRD patterns of the three TiO_2_ materials are shown in Figure 4. The peaks of m-TiO_2_ and n-TiO_2_ are situated at 25.3°, 37.8°, 48.0°, 53.8°, 55.0°, 62.6°, and 68.7°, which correspond to the (101), (004), (200), (105), (211), (204), and (116) crystallographic planes, respectively [35]. All the diffraction peaks were well established and perfectly matched to anatase TiO_2_ [39]. The intensity of the n-TiO_2_ diffraction peaks compares well to that of m-TiO_2_; however, the diffraction peaks are broader owing to the decrease in particle size [40]. The diffraction pattern of TNT is similar to the results obtained by other researchers, with two clear peaks located at 24.5° and 48.3° [41,42,43]. Harsha et al. [44] reported the existence of peaks that could be identified as titanate, which suggests that Na^+^ ions were replaced by H^+^ ions through ion exchange during the washing process. Zavala et al. [41] demonstrated that the anatase peaks did not fit well owing to the low crystallinity of the TNT. This was attributed to the collapse of the nanotubes during the ion exchange process or the formation of nanosheets as a precursor to the nanotubes.

**Figure 5:**
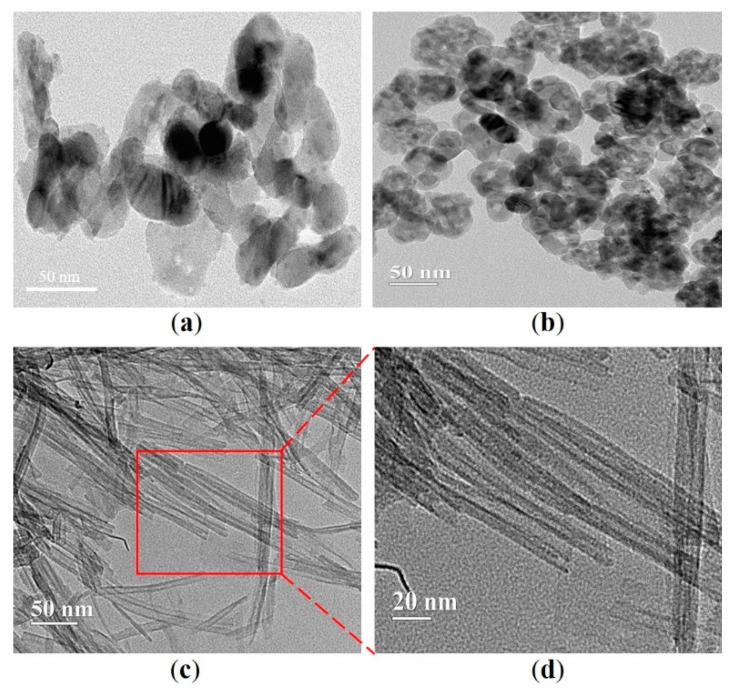
Morphology of (**a**) m-TiO_2_, (**b**) n-TiO_2_, and (**c**) TNT. (**d**) Higher magnification image of TNT.

**Figure 6:**
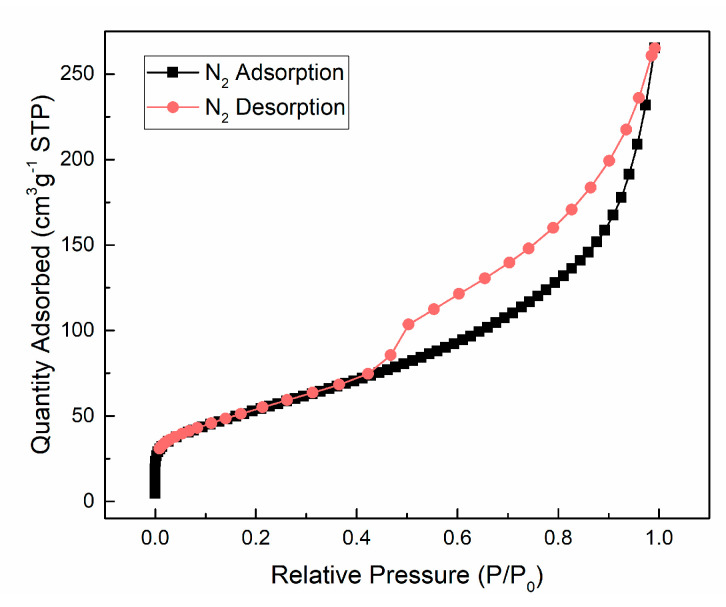
N_2_ adsorption isotherm of hydrothermally synthesized TNT.

Figure 5 shows the TEM micrographs of m-TiO_2_, n-TiO_2_, and TNT. The shape and the difference in size between the two TiO_2_ particles are clearly shown in Figure 5a,b, respectively. In addition, clusters or aggregates of particles composed of smaller particles are observed [45]. Figure 5c,d illustrate the structure of the TNT synthesized through the hydrothermal method, and the nanotubular structure of the TNT is apparent, and the nanotubes are hollow and open-ended [46]. This is a typical characteristic that could be used to identify TNT [47]. The size of the TNT is summarized in Table 4; the synthesized TNT exhibited an average length of 105 nm. The N_2_ adsorption isotherm of hydrothermally synthesized TNT is shown in Figure 6, and the curve was the hysteresis loop with a sharp inflection in N_2_ adsorbed volume at P/P_0_ was about 0.45, which suggested that the TNT was presented in tubular structures. The surface area of TNT obtained by BET was 196 m^2^/g. Compared with the physical properties of nano-TiO_2_ (45–55 m^2^/g), as shown in Table 2, TNT had an approximately four-fold larger surface area.

### 3.2. Hydration Products

**Figure 7:**
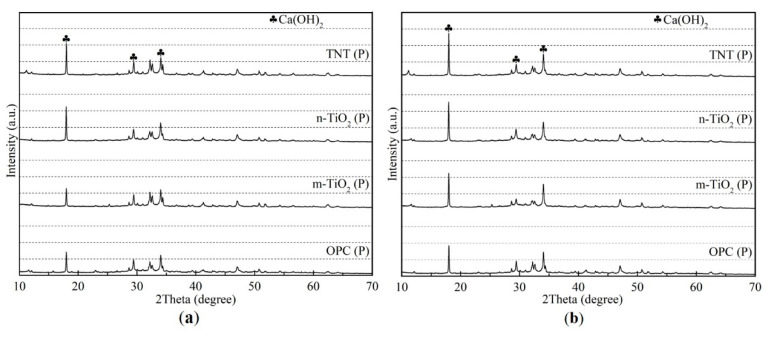
XRD patterns of the cement pastes after (**a**) 1 day and (**b**) 28 days of curing.

**Figure 8:**
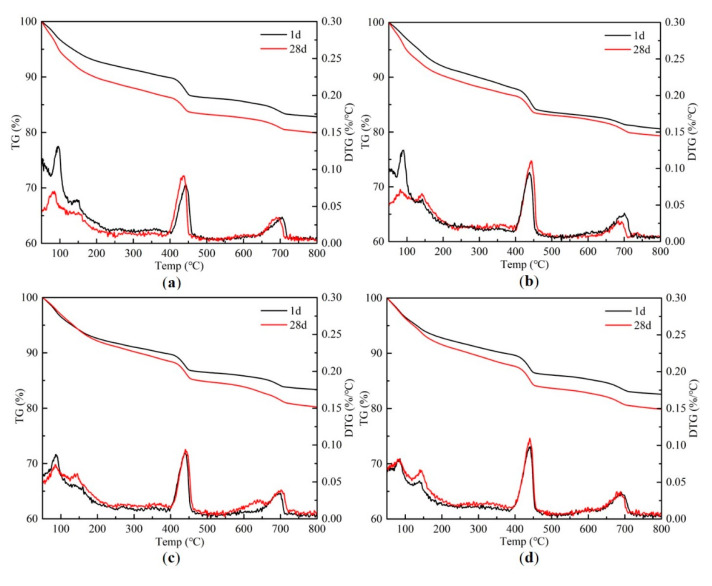
TG results of (**a**) OPC (P), (**b**) m-TiO_2_ (P), (**c**) n-TiO_2_ (P), and (**d**) TNT (P).

To further characterize the influence of the different TiO_2_ powders and TNT on the hydration of the cement paste, hydration product analysis was performed on the cement pastes utilizing XRD and TG. Figure 7 shows the XRD patterns of the OPC and cement pastes containing the different TiO_2_-based materials after 1 day and 28 days. The three main peaks of Ca(OH)_2_ appear at 2θ = 18.0°, 28.6°, and 34.1°. After 1 day of hydration, n-TiO_2_ and TNT accelerated the cement hydration because of their high surface areas providing nucleation sites for the hydration products, as previously reported [18,20]; this is in contrast to m-TiO_2_, which could be due to the differences in the sizes of the nanoparticles. After 28 days of hydration, the diffraction peaks attributed to Ca(OH)_2_ in all the XRD patterns of the cement pastes increased in intensity due to further hydration. In addition, the increase in the intensity of the Ca(OH)_2_ peaks was more evident in the hardened cement paste containing the TiO_2_ materials compared to OPC, especially for the hardened cement paste containing the TNT. This was possibly caused by the pronounced hollow tubular structure of the TNT compared to the TiO_2_ particles, which provides more locations and nucleation sites for the formation of hydration products, further allowing more hydration products to be generated [35]. This was also reflected in the TG results of the hardened cement pastes (Figure 8). When the hydrated cement pastes are exposed to high temperatures, mass loss occurs at specific temperature boundaries due to the loss of free water, dehydration, dehydrogenation, and decarbonization of the hydration products [48]. In summary, the thermal decomposition of hydration products was classified into main stages as follows: the weight loss around 100 °C corresponds to some of the cement hydration products, such as C–S–H, ettringite and monosulfate, etc., lose part of chemically bound water; the weight loss at 400–450 °C corresponds to the decomposition of Ca(OH)_2_; the weight loss above 600 °C corresponds to the decomposition of CaCO_3_ into CaO and CO_2_ [49]. It is evident from the TG results that the amount of Ca(OH)_2_ produced increased in the cement paste containing TNT, whether at 1 or 28 days, compared to the others. This result implies that TNT has great potential to improve the mechanical properties of cement pastes by accelerating the hydration of cement clinker phases in early and long-term aging.

### 3.3. Photocatalytic Performance

**Figure 9:**
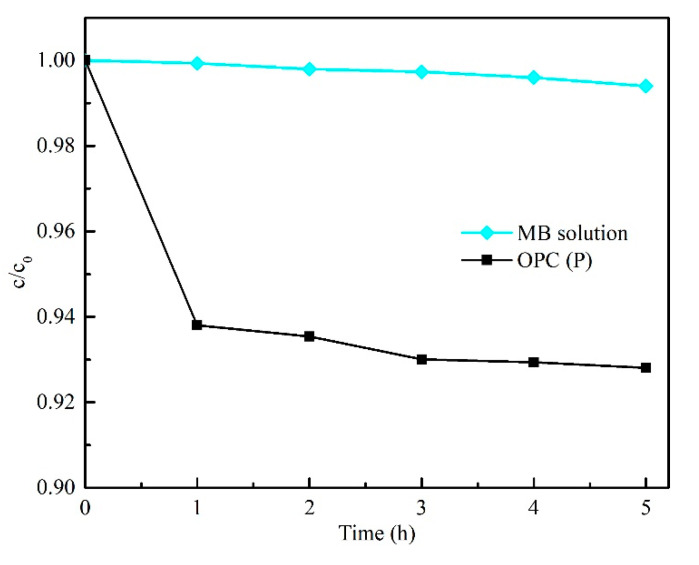
Concentration change of MB solution with and without OPC (P) under dark conditions.

According to other research, the concentration of MB solution decreased when cement paste was placed in MB solution; this may be attributed to the self-decomposition of MB and absorption by the cement paste [12,50,51]. Therefore, the change in concentration of MB solution with OPC and without OPC in dark conditions was measured, as shown in Figure 9. In our study, within 1 h, a decrease of MB concentration was found in OPC due to the porous structure of the OPC surface, whereas no significant self-decomposition of MB was found in pure MB solution.

**Figure 10:**
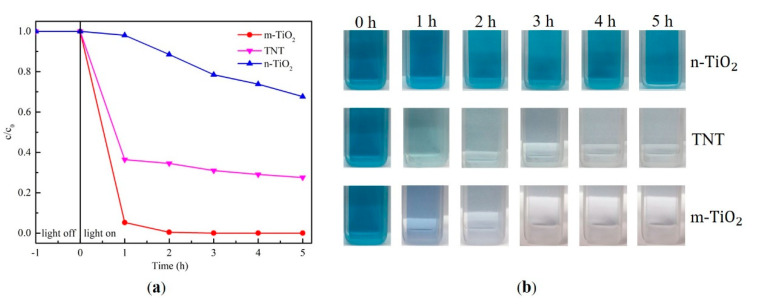
(**a**) Normalized concentration of methylene blue (MB) solution with pure photocatalysts and (**b**) the color change of the MB solution.

Figure 10a shows the photocatalytic properties of the three pure TiO_2_ photocatalysts (m-TiO_2_, n-TiO_2_, and TNT) in the degradation of MB. The data of decomposition of MB solution with pure photocatalysts is shown in Table 5. The concentration of the solution was almost unchanged in the dark condition (light off). The results indicate that m-TiO_2_ exhibits the best photocatalytic performance over 5 h. Figure 10b shows the dye photodegradation behavior as it discolors from dark blue to nearly transparent, especially in the presence of TNT and m-TiO_2_. The concentration of MB solution with TNT and m-TiO_2_ rapidly decreased after the first hour of UV irradiation, and then the solution concentration further decreased during the following 4 h.

**Figure 11:**
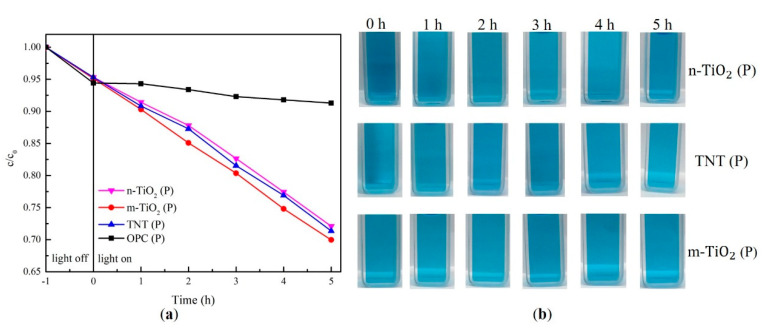
(**a**) Normalized concentration of MB solution with cement paste containing photocatalysts and (**b**) the color change of the MB solution.

In contrast, when the TiO_2_ photocatalyst was added to the cement (Figure 11), there was a significant decrease in the photocatalytic effect due to the lack of direct contact between the photocatalyst and the organic pollutants. The decomposition of the MB solution with cement paste samples is shown in Table 6. Before UV irradiation, the rapid decrease in the concentration of MB solution under dark conditions was due to the absorption of MB on the surface of porous OPC, as already observed in Figure 9. The normalized concentration of TNT (P) was lower than n-TiO_2_ (P); however, it was higher than that of m-TiO_2_ (P). This result was similar to that observed in the experiments conducted using only the photocatalysts (Figure 10). This is possibly owing to the hollow tubular structure of TNT, which provides a shorter and broader diffusion path for the dye contaminants from the solution to enter the reactive area of the photocatalyst compared to n-TiO_2_ [52]. m-TiO_2_ showed the best results. This is similar to the results reported by Folli et al. [53,54], which they attributed to two factors: first, m-TiO_2_ is more easily dispersed in the cement paste than the nanosized materials (TNT or n-TiO_2_), which are more significantly agglomerated and difficult to disperse by physical stirring when mixed with cement. Second, large molecules, such as MB, cannot easily penetrate the cement, and thus the well-dispersed m-TiO_2_ provides more available surface areas to adsorb and react with the large organic pollutants.

Recently, researchers have combined photocatalytic properties with cement-based materials by coating method. Feng et al. [14,55] fabricated TiO_2_ film on the cement paste surface utilizing the smear and spray method and found that the cement paste with the TiO_2_ was capable of degrading almost all of the dye solution. Shen et al. [56] prepared the concrete with an ultra-smooth surface covered with nano-TiO_2_ and tested the photocatalytic effect, and the result showed that the degradation rate of MB solution increased with increasing UV irradiation. Although some other studies using the coating method showed a better photocatalytic effect than our results, TNT fully has potential as a reinforcing material since it not only provides a photocatalytic effect on the cement material but also improves the mechanical strength of cement paste owing to its large aspect ratio. In this study, to quantitatively evaluate the effect of the TiO_2_ particles and nanotubes on the photocatalytic performance of cement paste, a specimen with a flat surface was utilized. To further improve the ability of the paste to degrade organic dyes, the contact surface area of the pastes could be increased. In this case, the use of TNT, which can improve the tensile strength of the cement paste [35], would be beneficial.

## 4. Conclusions

This study aimed to investigate the photocatalytic performance of TNT-reinforced cement paste and cement pastes modified with m-TiO_2_ and n-TiO_2_ for comparison. The TNT used in our work was prepared by the hydrothermal method using anatase TiO_2_ as the raw material. From the TEM results, it was evident that a hollow, open-ended, tubular structure was successfully synthesized. The XRD and TG results indicated that the TNT provided the most significant contribution to the hydration process of the cement, which can be beneficial to the mechanical development of cement pastes. After the same curing time, the TNT-reinforced cement paste produced the largest amount of hydration products. Regarding the photocatalytic performance, due to the wrapping of the hardening cement around the photocatalyst, the photocatalytic effects of the TNT-reinforced cement paste and the other two modified cement pastes were more limited than that of the photocatalysts alone. The cement paste containing m-TiO_2_ still showed the best capacity for photocatalytic degradation of MB solution, with about 30% of the MB solution was degraded. The TNT-reinforced cement paste followed closely behind. In summary, TNT-reinforced cement paste was able to produce more hydration products during the hydration process. Simultaneously, the tubular structure of TNT provided a better photocatalytic effect than n-TiO_2_, which is commonly used in cement photocatalytic studies. This offers valuable support for further research on the application of TNT in environmentally friendly cementitious materials.

## Figures and Tables

**Table 1:** Chemical composition (%) of ordinary Portland cement (OPC).

SiO_2_	Al_2_O_3_	Fe_2_O_3_	CaO	MgO	K_2_O	SO_3_	TiO_2_	LOI	Total
18.43	2.83	2.17	68.17	2.37	1.11	3.03	0.15	1.72	100

LOI: loss of ignition.

**Table 2:** Physical properties of anatase TiO_2_ powders.

Name	Particle Size	Surface Area	Purity
m-TiO_2_	0.15–0.25 µm	10.89 m^2^/g	98.5%
n-TiO_2_	<25 nm	45–55 m^2^/g	99.7%

**Table 3:** Mix proportion of cement paste.

Samples	m-TiO_2_ (g)	n-TiO_2_ (g)	TNT (g)	W/C	Water (g)	Cement (g)
OPC (P)	-	-	-	0.4	16	40
m-TiO_2_ (P)	0.4	-	-			
n-TiO_2_ (P)	-	0.4	-			
TNT (P)	-	-	0.4			

(P) in the sample name denotes cement pastes.

**Table 4:** The size of TNT synthesized by the hydrothermal method.

Product	Outside Diameter	Inside Diameter	Average Length	Max Length	Min Length	Surface Area
TNT	8 ± 3.4 nm	3 ± 2.5 nm	105 nm	200 nm	50 nm	196 m^2^/g

**Table 5:** The decomposition of MB solution with pure photocatalysts.

	Concentration of MB Solution (mg/mL)					
	0 h	1 h	2 h	3 h	4 h	5 h
m-TiO_2_	1.51	0.02	0.00	0.00	0.00	0.00
n-TiO_2_	1.51	1.32	1.18	1.13	1.05	0.97
TNT	1.51	0.51	0.5	0.45	0.43	0.40

**Table 6:** The decomposition of MB solution with cement paste samples.

	Concentration of MB Solution (mg/mL)					
	0 h	1 h	2 h	3 h	4 h	5 h
OPC (P)	1.41	1.41	1.39	1.38	1.37	1.36
m-TiO_2_ (P)	1.42	1.32	1.24	1.15	1.09	1.03
n-TiO_2_ (P)	1.42	1.39	1.33	1.25	1.17	1.08
TNT (P)	1.42	1.37	1.32	1.23	1.16	1.09
